# Protein Solubility, Digestibility and Fractionation after Germination of Sorghum Varieties

**DOI:** 10.1371/journal.pone.0031154

**Published:** 2012-02-03

**Authors:** Abd El-Moneim M. R. Afify, Hossam S. El-Beltagi, Samiha M. Abd El-Salam, Azza A. Omran

**Affiliations:** 1 Department of Biochemistry, Faculty of Agriculture, Cairo University, Giza, Cairo, Egypt; 2 Department of Crops Technology, Food Technology Research Institute, Agricultural Research Center, Cairo, Egypt; University of South Florida College of Medicine, United States of America

## Abstract

The changes in crude protein, free amino acids, amino acid composition, protein solubility, protein fractionation and protein digestibility after germination of sorghum were investigated. Sorghum varieties (Dorado, Shandaweel-6, Giza-15) were soaked for 20 h followed by germination for 72 h; the results revealed that crude protein and free amino acids in raw sorghum varieties ranged from 10.62 to 12.46% and 0.66 to 1.03 mg/g, respectively. Shandaweel-6 was the highest variety in crude protein and free amino acids content. After germination, crude protein was decreased and free amino acids were increased. There was an increase in content of valine and phenylalanine amino acids after germination. On the other hand, there was a decrease in most of amino acids after germination. After germination protein solubility was significantly increased. Regarding protein fractions, there was an increase in albumin, globulin and kafirin proteins and a decrease in cross linked kafirin and cross linked glutelin after germination.

## Introduction

Sorghum (*Sorghum bicolor* [L.] Moench) one of the most important weaning foods in low-income and high-income countries [1]–[5]. It ranks fifth among the world cereals, following wheat, maize, rice and barley in production area and total production. Sorghum is an extremely important crop in Asia, Africa and other semi-arid regions of the world [6].

The nutrient composition of sorghum indicates that it is a good source of energy, proteins, carbohydrates, vitamins and minerals [7]–[9].

Sorghum components, especially its protein is less digestible than other cereals for human and monogastric animals, because of its anti-nutritional factors such as tannins and phytic acid. Removal of these undesirable components is essential to improve the nutritional quality of sorghum and effectively utilize its potential as human food or animal feed [10], [11].

Interaction between tannins and sorghum proteins reduces both protein and starch digestibility. This is important in both human and animal nutrition. The formation of complexes between sorghum proteins and tannins is thought to render the proteins indigestible as well as inhibit digestive enzymes. Proteins rich in proline bind more sorghum tannins than other proteins. In addition, a protein containing more proline repeats will bind more tannin than one with less such repeats [12].

The low digestibility of sorghum proteins is presumably due to the high protein cross linking. Good quality proteins are those that are readily digestible and contain the essential amino acids in quantities that correspond to human requirements [13]–[18].

The *in vitro* pepsin digestion assay is mimics the digestive system, and are widely used to study the structural changes, digestibility, and release of food components under simulated gastrointestinal conditions. The most frequently used biological molecules included in the digestion models were digestive enzymes, bile salts, and mucin [19], [20].

Germination is widely used in legumes and cereals to increase their palatability and nutritional value, particularly through the breakdown of certain antinutrients, such as phytate and protease inhibitors [21], [22].

Germination is a common practice in sorghum producing areas. Grains are malted for the production of weaning foods, opaque beers and other traditional dishes. Germination triggers the enzymatic activity of sprouting grains, leading to the breakdown of proteins, carbohydrates and lipids into simpler forms. This processing method activates proteases which are active in degrading proteins, thereby increasing nutrient bioavailability [23].

The objective of this study was to enhance sorghum nutritional value *via* germination, identify of sorghum protein characteristics, such as protein digestibility, protein solubility and protein fractionation as well as amino acids contents.

## Materials and Methods

### Materials

#### Samples and chemicals

Pepsin, pancreatin, α-amylase and L-aspartic acid were obtained from Sigma– Aldrich Chemical Co., St. Louis, USA. All other chemicals used were of analytical reagent grade.

Three white sorghum varieties (*Sorghum bicolor* L. Moench), Shandaweel-6 was obtained from the Crops Research Institute, Agricultural Research Centre, and Dorado and Giza-15 were obtained from Central Administration for Seed Certification (CASC), Ministry of Agriculture and Land Reclamation, Giza, Egypt. The grains were carefully cleaned and freed from broken grains and extraneous matter.

#### Germination of grains

Sorghum grains were soaked in distilled water for 20 h with a ratio 1∶5 w/v and the soaked water changed twice. At the end of soaking period, the soaked water was discarded. Soaked grains were germinated for 72 hours at room temperature, and then the grains were dried. The root and shoot portions were manually removed. The grains were milled into fine flour and kept until analysis.

### Chemical analysis

#### Determination of crude protein

Crude protein contents of raw sorghum and treatments were determined according to the methods of A.O.A.C. [24].

#### Determination of free amino acids

Free amino acids were determined using the method outlined by Rosen [25]. Ninhydrin reagent used for the determination of free amino acids. The free amino acids were calculated as mg/g DW from the standard curve which prepared by using L-aspartic acid as standard.

#### Determination of amino acids composition

Amino acids composition of samples was determined by using amino acid analyzer (Biochrom 30) according to the method outlined in A.O.A.C. [24]. An aliquot sample, were weighed and digested with 25 ml of 6N HCl at 110°C for 24 h. Then HCl was removed by evaporation; the remaining solid fraction was dissolved with 0.2N sodium citrate buffer (pH 2.2). One ml of the solution was filtered though 0.45 µm Millipore membrane filters. The standard amino acids (consist of 17 amino acids) was treated as the same as of the samples. Amino acids were expressed as g/100 g protein on dry weight basis.

#### Determination of protein solubility

Protein solubility was determined by the method of Sathe and Salunkhe [26]. One gram of samples was dispersed in 25 ml of 1 M NaOH. The obtained suspensions were mixed and stirred in an orbital shaker at 150 rpm for 12 h at room temperature and then centrifuged at 3000 g for 20 min. Soluble proteins in supernatants were determined by Lowry *et al.* [27]. Bovine Serum Albumin was used as standard protein. Soluble protein was expressed as g/100 g DW sample.

#### Determination of sorghum protein fractions

Protein fractionation was performed according to the method modified from Landry and Moureaux [28]. Two grams of sorghum were sequentially extracted with the six solvents listed below (20 ml at 25°C and centrifuged at 18,900 g for 10 min at 4°C after each step). Initially, sorghum was extracted with deionized water for 20 min (albumins, fraction 1) and the pellet sequentially extracted with the following solutions: 0.5 M NaCl for 60 min (globulins, fraction 2); 60% 2-propanol (v/v) for 4 h (kafirins, fraction 3); 0.1 M borate buffer, pH 10.8, for 4 h (glutelin-like protein, fraction 4); 60% 2-propanol with 1% dithiothreitol (DTT) for 4 h (cross-linked kafirins, fraction 5); and 0.1 M borate buffer, pH 10.8, containing 1% DTT and 1% sodium dodecyl sulfate (SDS) for 18 h at 4°C (cross-linked glutelins, fraction 6). Soluble protein in supernatants was determined by Lowry *et al.* [27] for fractions 1 to 4 and by de Wreede and Stegemann [29] from fractions 5 and 6. Protein fractions were expressed as g/100 g protein on dry weight basis.

#### Determination of in vitro protein digestibility

*In vitro* protein digestibility was determined according to the method of Akeson and Stahmanna [30]. One gram samples added to HCl (15 ml, 0.1 M), containing 1.5 mg pepsin then the incubated at 37°C for 3 h. The obtained suspension was neutralized with NaOH (7.5 ml, 0.2 M), then treated with 4 mg of pancreatin in 7.5 ml 0.2 M phosphate buffer (pH 8.0). One milliliter of toluene was added to prevent microbial growth and the mixture was gently shaken and incubated for additional 24 h at 37°C. After incubation, the sample was treated with 10 ml of 10% TCA to remove undigested protein and larger peptides and centrifuged at 50000 g for 20 min at room temperature. Protein in the supernatant was estimated using the Kjeldahl method (A.O.A.C.) [24]. The percentage of protein digestibility was calculated by the ratio of protein in supernatant to protein in sample as equation:
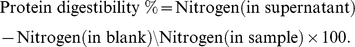



#### Statistic analysis

For the analytical data, mean values and standard deviation are reported. The data obtained were subjected to one-way analysis of variance (ANOVA) and least significant difference (LSD) at *P*<0.05.

## Results and Discussion

### Effect of germination of sorghum on crude protein and free amino acids

Table 1 presents crude protein and free amino acids content in sorghum before and after germination. Protein content ranged from 10.62 to 12.46% in raw sorghum varieties. Protein was significantly higher in Shandaweel-6 while Giza-15 was the lowest one. These results are in agreement with Dicko *et al.* [7] and Johnson *et al.* [31] who found that crude protein content in whole sorghum grain is ranged from 7 to 15% or 10.30 to 14.90%.

**Table 1:** Crude protein and free amino acids content of sorghum after germination.

Treatments	Crude protein (%)	Free amino acids (mg/g DW)*
**Raw**		
**Dorado**	10.90±0.14^c^	0.66±0.01^e^
**Shandaweel-6**	12.46±0.11^a^	1.03±0.03^c^
**Giza-15**	10.62±0.20^d^	0.82±0.03^d^
**Germination**		
**Dorado**	10.25±0.20^e^	8.78±0.05^b^
**Shandaweel-6**	12.10±0.10^b^	9.94±0.04^a^
**Giza-15**	9.77±0.09^f^	8.80±0.04^b^
**L.S.D**	0.2632	0.0561

*mg/g DW = mg per gram dry weight.

Values are mean of three replicates ±SD, number in the same column followed by the same letter are not significantly different at *p*<0.05 level.

The crude protein was significantly decreased after germination compared with raw sorghum. These results are agreed with Shaker *et al.* [32] who reported that nutrients loss might be attributed to the leaching of soluble nitrogen, mineral and other nutrients into desired solution.

Free amino acids content ranged from 0.66 to 1.03 mg/g in raw sorghum. Free amino acids amount was significantly higher in Shandaweel-6 than other varieties. After germination free amino acids content were increased and ranged from 8.78 to 9.94 mg/g and this may be due to the activity of proteolytic enzymes. Chavan *et al.* [33] found that free amino acids content in germinated sorghum (72 h) was 1.20 mg/g.

The nutritive value of food, especially protein mostly would depend not only on its amino acid profile in general but also on the quantities of the essential amino acids content in particular. The nutritive value of dietary protein is determined by the pattern and quantity of essential amino acids present.

Table 2 shows essential amino acids content in sorghum before and after germination. Essential amino acids in raw sorghum ranged from 3.49 to 3.58, 11.74 to 13.56, 2.75 to 3.21, 4.22 to 4.43, 4.40 to 4.98, 2.11 to 2.26, 2.73 to 2.94 and 4.22 to 4.58 g/100 g protein for isoleucine, leucine, threonine, valine, phenylalanin, lysine, methionine and tyrosine, respectively. Shandaweel-6 had the highest amount of isoleucine, leucine, threonine, valine, phenylalanin and tyrosine, respectively. While Dorado had the highest amount of methionine. Finely, Giza-15 had the highest amount of lysine. The results are in the same trend with Mokrane *et al.* [34] who reported that essential amino acids content in raw sorghum ranged from 3.69 to 4.27, 15.77 to 17.34, 4.47 to 5.07, 4.62 to 5.11, 4.49 to 6.26, 1.58 to 2.05, 1.06 to 1.42 and 2.69 to 3.19 g/100 g protein for isoleucine, leucine, threonine, valine, phenylalanin, lysine, methionine and tyrosine, respectively. Moreover, essential amino acids content in raw sorghum ranged from 3.64 to 3.71, 11.50 to 13.37, 2.99 to 3.45, 4.64 to 5.90, 4.54 to 4.81, 1.66 to 1.88, 1.00 to 1.76 and 3.53 to 3.76 g/100 g protein for isoleucine, leucine, threonine, valine, phenylalanin, lysine, methionine and tyrosine, respectively [35].

**Table 2:** Essential amino acids content of sorghum after germination (g/100 g protein).

Treatments	Isoleucine	Leucine	Threonine	Valine	Phenylalanine	Lysine	Methionine	Tyrosine
**Raw**								
**Dorado**	3.49±0.01^d^	11.74±0.01^f^	2.75±0.01^d^	4.22±0.02^f^	4.40±0.01^e^	2.11±0.01^d^	2.94±0.02^a^	4.22±0.02^d^
**Shandaweel-6**	3.85±0.01^a^	13.56±0.02^a^	3.21±0.01^a^	4.82±0.03^d^	4.98±0.01^c^	2.17±0.02^c^	2.73±0.01^c^	4.22±0.02^d^
**Giza-15**	3.58±0.01^c^	11.96±0.03^c^	2.92±0.01^b^	4.43±0.01^e^	4.61±0.01^d^	2.26±0.02^b^	2.82±0.01^b^	4.33±0.01^b^
**Germination**								
**Dorado**	3.42±0.02^e^	12.00±0.01^b^	2.54±0.02^e^	5.85±0.05^a^	4.00±0.03^f^	2.34±0.01^a^	2.44±0.01^e^	4.29±0.03^c^
**Shandaweel-6**	3.80±0.02^b^	11.82±0.01^e^	2.89±0.01^c^	4.96±0.02^c^	5.37±0.02^b^	1.98±0.02^f^	2.48±0.02^d^	4.13±0.01^e^
**Giza-15**	3.38±0.03^f^	11.87±0.01^d^	2.87±0.02^c^	5.32±0.02^b^	5.73±0.02^a^	2.05±0.03^e^	2.15±0.02^f^	4.20±0.01^d^
**L.S.D**	0.0325	0.0299	0.0252	0.0498	0.0325	0.0348	0.0281	0.0325

Values are mean of three replicates ±SD, number in the same column followed by the same letter are not significantly different at *p*<0.05 level.

From the data in Table 2, it could be noticed that after treatments essential amino acids content were changed. There was an increase in content of valine and phenylalanine after germination. On the other hand, there was a decrease in most of amino acids after germination. These findings are in agreement with Elemo *et al.* [36] who found that germination significantly increased the essential amino acids except for histidine and sulphur amino acids. Germination of cereals and legumes has been shown to be generally advantageous as it also improves the nutritional qualities of cereals and legumes [37].

Table 3 shows non essential amino acids content in sorghum before and after germination. Non essential amino acids content in raw sorghum ranged from 3.58 to 4.01, 6.24 to 7.06, 7.43 to 8.83, 6.66 to 8.99, 18.45 to 20.63, 2.84 to 3.05, 3.49 to 4.17 and 1.93 to 2.17 g/100 g protein for arginine, aspartic acid, alanine, prolin, glutamic acid, glycine, serine, histidine and cysteine. Shandaweel-6 had the highest amount of arginine, aspartic acid, alanine, glutamic acid, glycine, and serine. While Dorado had the highest amount of prolin and cystine. Also, Shandaweel-6 and Giza-15 had the same amounts of histidine. Glutamic acid was found to be the major non-essential amino acids in the tested samples, while cystine was the lowest one.

**Table 3:** Non essential amino acids content of sorghum after germination (g/100 g protein).

Treatments	Arginine	Aspartic	Alanine	Proline	Glutamic	Glycine	Serine	Histidine	Cysteine
**Raw**									
**Dorado**	3.58±0.02^c^	6.24±0.02^f^	7.43±0.01^e^	8.99±0.01^a^	18.45±0.05^d^	2.84±0.02^d^	3.49±0.01^d^	1.93±0.01^c^	2.11±0.01^a^
**Shandaweel-6**	4.01±0.01^a^	7.06±0.04^a^	8.83±0.02^a^	6.66±0.02^e^	20.63±0.03^b^	3.05±0.02^a^	4.17±0.03^a^	2.17±0.01^a^	1.69±0.02^d^
**Giza-15**	3.77±0.02^b^	6.87±0.03^b^	7.72±0.02^d^	7.53±0.02^d^	19.77±0.03^c^	2.92±0.01^b^	3.58±0.02^c^	2.17±0.02^a^	1.69±0.01^d^
**Germination**									
**Dorado**	3.22±0.02^e^	6.44±0.04^e^	8.20±0.04^c^	8.88±0.02^b^	20.78±0.02^a^	2.73±0.01^e^	3.32±0.01^e^	2.05±0.01^b^	2.05±0.02^b^
**Shandaweel-6**	3.47±0.03^d^	6.78±0.02^c^	6.94±0.02^f^	6.61±0.01^f^	19.75±0.05^c^	2.73±0.02^e^	3.80±0.02^b^	2.07±0.02^b^	1.65±0.01^e^
**Giza-15**	3.58±0.02^c^	6.65±0.01^d^	8.39±0.01^b^	7.68±0.02^c^	19.75±0.03^c^	2.87±0.01^c^	3.28±0.01^f^	2.05±0.01^b^	1.84±0.01^c^
**L.S.D**	0.0370	0.0513	0.0398	0.0308	0.0643	0.0281	0.0325	0.0252	0.0252

Values are mean of three replicates ±SD, number in the same column followed by the same letter are not significantly different at *p*<0.05 level.

The results are in the same trend with other study, which cited that non essential amino acids in raw sorghum ranged from 5.61 to 6.13, 7.75 to 9.39, 7.60 to 8.32, 16.44 to 16.89, 1.92 to 2.95, 3.12 to 4.04 and 1.90 to 2.24 g/100 g protein for aspartic acid, alanine, prolin, glutamic acid, glycine, serine and histidine, respectively [38]. Essential amino acids content in raw sorghum ranged from 7.21 to 7.58, 7.29 to 9.19, 20.03 to 23.16, 2.58 to 2.92, 1.73 to 2.60, 4.44 to 4.73 and 1.89 to 2.96 g/100 g protein for aspartic acid, proline, glutamic acid, glycine, cysteine, serine and histidine, respectively [35].

After germination, most of non essential amino acids content was decreased. Moreover, proline content was decreased except for germinated Giza-15 which seems to be significantly increased than raw Giza-15. The breakdown of protease resistant prolamins and the increase of essential amino acids upon germination have been reported [38].

### Effect of germination of sorghum on protein solubility and Protein fractions

Among the functional properties of proteins, solubility is probably the most critical function. Protein solubility characteristics are influenced by factors such as origin, processing conditions, pH, ionic strength and the presence of other ingredients [39], [40].

Table 4 exhibits the effect of germination of sorghum on protein solubilized under alkaline conditions extracted with NaOH as described in materials and methods. Data showed that solubility of raw sorghum protein ranged from 3.21 to 3.72 g/100 g. Shandaweel-6 recorded the highest variety in protein solubility.

**Table 4:** Protein solubility of sorghum after germination (g/100 g DW)*.

Treatments	Protein solubility
**Raw**	
**Dorado**	3.45±0.08^de^
**Shandaweel-6**	3.72±0.32^cd^
**Giza-15**	3.21±0.13^e^
**Germination**	
**Dorado**	4.11±0.08^b^
**Shandaweel-6**	4.90±0.06^a^
**Giza-15**	3.98±0.33^bc^
**L.S.D**	0.3597

*mg/g DW = mg per gram dry weight.

Values are mean of three replicates ±SD, number in the same column followed by the same letter are not significantly different at *p*<0.05 level.

There was a significant increase in protein solubility after germination. These findings are in agreement with Elkhalifa and Bernhardt [40], who found that germinated sorghum had a higher protein solubility compared with the un-geminated one. The protein of the germinated sorghum was more soluble than the un-geminated sorghum. This might be due to the high proteolytic activity during germination, which will lead to an increase in the protein solubility resulting from hydrolysis of the storage proteins.

The proteins of the sorghum grain are classically divided, based on solubility in different solvents: water-soluble (albumins), salt-soluble (globulins), aqueous alcohol-soluble (kafirins), aqueous alcohol reducing agent-soluble (cross-linked kafirins) and detergent reducing agent alkaline pH-soluble (cross-linked glutelins) [41].

**Figure 1:**
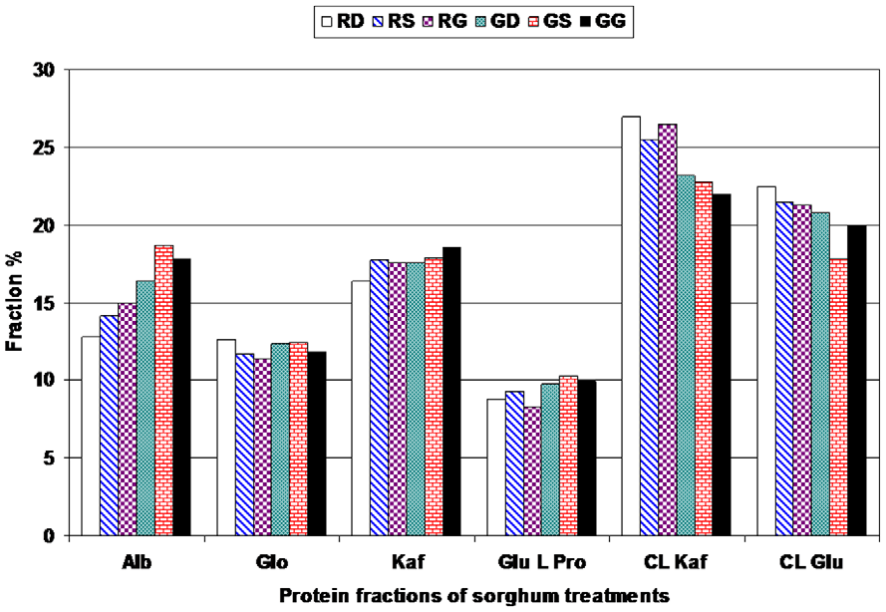
Protein fractions of sorghum after germination (Alb: Albumin; Glo: Globulin; Kaf: Kafirin; Glu L Pro: Glutelins like protein; CL Kaf: Cross linked kafirins; CL Glu: Cross linked glutelins). Treatments: (RD: Raw Dorado; RS: Raw Shandweel-6; RG: Raw Giza-15; GD: Germinated Dorado; GS: Germinated Shandaweel-6; GG: Germinated Giza-15).

Figure 1 presents the effect of germination treatment on protein fractions based on solubility for each fraction, into albumins, globulins, kafirins, glutelins like protein, cross linked kafirin and cross linked glutelins. From results, it could be noticed that raw sorghum contain 12.77 to 14.95%, 11.37 to 12.67%, 16.37 to 17.75%, 8.30 to 9.35, 25.55 to 27.00% and 21.31 to 22.45% for albumins, globulins, prolamins, glutline like protein, cross linked kafirins and cross linked glutline, respectively. Distribution of protein in fractions extracted with the different solvents suggested that, the three sorghum varieties different in amount of total extractable protein and this is may be due to the differences in total protein. Cross linked kafirins, represented a considerably greater fraction in sorghum varieties. Results are close to Ejeta *et al.* [42], who found that fractionated protein in raw sorghum ranged from 10.00 to 24.00%, 6 to 16% and 11.00 to 31.00% for albumins plus globulins, prolamins and cross linked kafirins, respectively. Raw corn contain 19.50 to 26.20%, 20.90 to 35.30% and 15.20 to 23.80% for albumins plus globulins, cross linked kafirins and cross linked glutline, respectively [43]. Sorghum prolamins ranged from 42.50 to 81.80% [30]. Since a large percentage of sorghum kafirin storage proteins exist in polymeric forms linked by disulfide bonds in their native state, differences in content of fractions rich in insoluble disulfide proteins, i.e., cross-linked kafirin and cross linked glutelin could contribute to protein digestibility differences. Moreover, kafirins and cross linked kafirin in raw sorghum ranged from 17.30 to 19.90% and 24.50 to 35.10, respectively [44]. Sorghum has a higher proportion of cross linked prolamin than maize [45].

The results revealed an increase in albumin, globulin and kafirin fractions and a decrease in cross linked kafirin and cross linked glutelin after germination. These results are close to previous studies, which found that albumin, globulin and glutelin like protein were increased and prolamins, cross linked kafirin and cross linked glutelin fraction were decreased after germination [46], [47].

### Effect of germination of sorghum on *in vitro* protein digestibility (IVPD)

The factors that may affect sorghum protein digestibility, divided in two categories: exogenous factors (*i.e.* interactions of proteins with non-protein components such as polyphenols, starch, non-starch polysaccharides, phytates and lipids), and endogenous factors (factors arising from the sorghum proteins themselves), concluding that the poor digestibility of sorghum proteins appear to be multi-factorial. The main proteins in sorghum were kafirins. These proteins are known to be peptidase resistant because of their S-S bonds. Digestibility may be used as an indicator of protein availability [13], [47].

**Figure 2:**
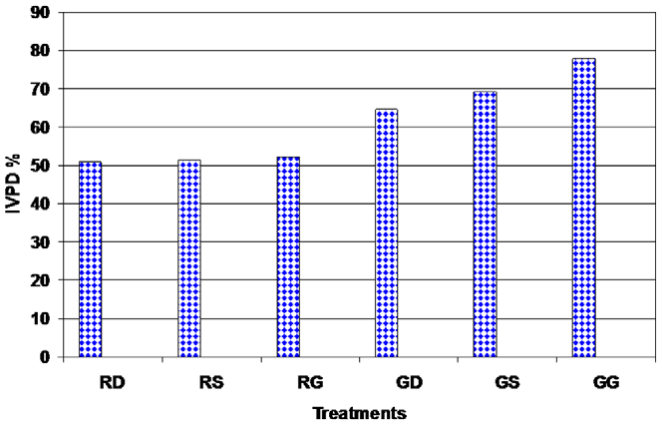
*In vitro* protein digestibility (IVPD%) of sorghum after germination. Treatments: (RD: Raw Dorado; RS: Raw Shandweel-6; RG: Raw Giza-15; GD: Germinated Dorado; GS: Germinated Shandaweel-6; GG: Germinated Giza-15).

Figure 2 presents the *in vitro* protein digestibility in sorghum before and after germination. Data showed that *in vitro* protein digestibility in raw sorghum ranged from 50.94 to 52.09% and Giza-15 was significantly higher in protein digestibility than other varieties. The findings are in agreement with Zhao *et al.* [13], who showed that the *in vitro* protein digestibility ranged from 23.00 to 68.20%. The low digestibility of sorghum proteins is presumably due to the high protein cross-linking.

In addition, *in vitro* protein digestibility was significantly increased after germination treatments. Germinated Giza-15 was the highest variety in protein digestibility compared with other varieties. These results are similar to Correia *et al.* [48], who found that germination causes activation of intrinsic amylases, proteases, phytases and fiber-degrading enzymes, thereby increasing nutrient digestibility. The activity of intrinsic proteases in germinated grains leads to an increase in *in vitro* protein digestibility. Germination is effective in increasing protein digestibility and improving sensory properties Also, processing of sorghum (boiling, germination, fermentation and cooking) greatly improved its nutritive value [49], [50].
