# Expression of concern: Protein solubility, digestibility and fractionation after germination of sorghum varieties

**DOI:** 10.1371/journal.pone.0351579

**Published:** 2026-06-15

**Authors:** The PLOS One Editors

The PLOS One Editors issue this Expression of Concern for this article [1] because of concerns identified about the peer review process. Readers are advised to interpret the article [1] with caution.
